# Effects of different kinds of essentiality on sequence evolution of human testis proteins

**DOI:** 10.1038/srep43534

**Published:** 2017-03-08

**Authors:** Julia Schumacher, Hans Zischler, Holger Herlyn

**Affiliations:** 1 Institute of Anthropology, Johannes Gutenberg University Mainz, Anselm-Franz-von-Bentzel-Weg 7, D-55099, Germany

## Abstract

We asked if essentiality for either fertility or viability differentially affects sequence evolution of human testis proteins. Based on murine knockout data, we classified a set of 965 proteins expressed in human seminiferous tubules into three categories: proteins essential for prepubertal survival (“lethality proteins”), associated with male sub- or infertility (“male sub-/infertility proteins”), and nonessential proteins. In our testis protein dataset, lethality genes evolved significantly slower than nonessential and male sub-/infertility genes, which is in line with other authors’ findings. Using tissue specificity, connectivity in the protein-protein interaction (PPI) network, and multifunctionality as proxies for evolutionary constraints, we found that of the three categories, proteins linked to male sub- or infertility are least constrained. Lethality proteins, on the other hand, are characterized by broad expression, many PPI partners, and high multifunctionality, all of which points to strong evolutionary constraints. We conclude that compared with lethality proteins, those linked to male sub- or infertility are nonetheless indispensable, but evolve under more relaxed constraints. Finally, adaptive evolution in response to postmating sexual selection could further accelerate evolutionary rates of male sub- or infertility proteins expressed in human testis. These findings may become useful for *in silico* detection of human sub-/infertility genes.

Almost four decades ago, Wilson *et al*.^1^ hypothesized that proteins essential for an organism’s viability or fertility should evolve at lower rates than those which are more dispensable. This predicted association has been studied extensively in mammalian (see, e.g., refs 2, 3, 4) and non-mammalian species (see, e.g., refs 5, 6, 7), but the respective investigations yielded inconsistent outcomes. In the mentioned studies on mammals essentiality was defined as indispensability for either viability or both survival and fertility. In contrast, Torgerson *et al*.^8^ differentiated between fertility and viability proteins and reported that murine proteins important to either male or female reproduction evolve at higher rates than proteins indispensable for survival and also than a representative genomic sample. This observation is in line with previous studies’ findings whereupon reproduction-related proteins show accelerated rates of evolution^9^ and are oftentimes subject to positive selection^10^. In particular, male reproductive proteins have been described to evolve rapidly (see, e.g., refs 11 and 12). Furthermore, genes with testis-specific expression evolve at overall higher rates relative to female-specific genes or those unrelated to reproduction in *Drosophila*^13^ and relative to genes with expression maxima in other rodent tissues^14^. Thereby, rate acceleration of male reproductive proteins is assumed to be driven by different forms of postmating sexual selection, such as sperm competition and sexual conflict (see, e.g., refs 14 and 15). Although sexual selection could indeed explain enhanced evolutionary rates of some male reproductive proteins, sequences of the majority of proteins expressed in sperm or the male reproductive tract are evolutionarily conserved (see, e.g., refs 16 and 17). One possible explanation for this prevailing conservation may be proteins’ involvement in basic cellular functions such as metabolism^16^,^18^. But while the influence of sperm proteins’ functions on their evolutionary rate has already been explored (see, e.g., refs 18 and 19), the effects of different forms of essentiality, i.e. for viability or fertility, on evolutionary rates of male reproductive proteins have not yet been disentangled.

Therefore, the present study aimed to unveil the impact of different kinds of essentiality on sequence evolution of testis proteins. Our analyses were conducted on a sample of human testis proteins relying on protein expression data. We distinguished between proteins associated with prepubertal death (“lethality proteins”), linked to male sub- or infertility (“male sub-/infertility proteins”), and “nonessential proteins”, associated with neither form of essentiality. Testis proteins were assigned to one of these three categories based on known phenotypes resulting from targeted knockout (KO) mutations in murine orthologues of human genes. We hypothesized that lethality proteins evolve under stronger purifying selection due to their functional importance and increased evolutionary constraints. In contrast, relaxation of constraints as well as sexual selection might accelerate sequence evolution of more specialized^8^, but nonetheless important sub-/infertility proteins.

To assess levels of evolutionary constraints, we employed three measures. First, we derived node degree, the number of links a protein has to other nodes, from a human protein-protein interaction (PPI) network. Second, tissue specificity was estimated using the index τ, which ranges from 0 to 1, whereby higher values indicate more tissue-biased expression^20^. Third, numbers of biological processes in which a protein participates served as a measurement of its multifunctionality^21^. Numbers of PPI partners, expression breadth, and multifunctionality are known to correlate with pleiotropy^22^,^23^ and have been previously used to quantify levels of pleiotropy (see, e.g., refs 24 and 25). Moreover, PPIs also exert structural and functional constraints on proteins (see, e.g., refs 26, 27, 28). Hence, the applied properties enable assessment of a broad range of constraints under which proteins evolve. Magnitude and direction of selection were measured using pairwise dN/dS estimates between human and mouse orthologues. The dN/dS estimate contrasts nonsynonymous (dN) and synonymous substitution rates (dS). Thereby, dN/dS values >1, <1, and = 1 are associated with positive selection, purifying selection, and neutral evolution, respectively. Before comparing the three protein categories – lethality, sub-/infertility, and nonessential proteins – regarding their evolutionary rates and constraints, we evaluated the interrelations among dN/dS, node degree, multifunctionality, and tissue specificity within our human testis dataset employing rank correlations. In doing so, we were able to examine the interdependencies among essentiality, evolutionary constraints, and rates of sequence evolution in a set of human testis proteins.

## Results and Discussion

### Characteristics of the human testis protein dataset

The initial dataset contained 2,986 proteins expressed in human seminiferous tubules according to information from Human Protein Atlas version 12 (see Materials and Methods). Although testes consist of various cell and tissue types, we focused on proteins from seminiferous tubules including Sertoli cells as well as spermatogenesis and spermiogenesis stages. In the course of mapping IDs from Ensembl version 73 to 82 (see Materials and Methods), four of the 2,986 IDs were mapped to IDs already existing in the dataset. These redundant IDs were discarded. For further 602 genes, no dN/dS estimate calculated for human and murine 1-to-1 orthologues was available in Ensembl version 82. Another 344 proteins were not contained in the PPI network used (see Materials and Methods) and lack of murine KO data (see Materials and Methods) led to exclusion of 964 proteins. Finally, five proteins were excluded due to lacking gene ontology (GO) biological process data (see Materials and Methods). For 1,067 proteins all of the collected variables including murine phenotype data for targeted KO mutations were available. Of these, 73 were excluded because their ablation in mice resulted in both male reproductive anomalies and prepubertal lethality. Another 26 proteins were removed from the dataset because their KO mutants had an unclear fertility status, due to difficult transferability of the observed phenotype to humans (“decreased litter size”), or because male null mice were fertile despite reproductive abnormalities (for details, see Materials and Methods). Finally, three more proteins were excepted from analyses due to further reasons which are outlined in detail in the Materials and Methods section. After exclusion of these overall 102 proteins, the final dataset contained 965 testis proteins, comprising 57 male sub-/infertility, 502 lethality, and 406 nonessential proteins (for 965 included and 102 excluded proteins, see Supplementary Table S1).

None of the included 965 genes coding for human testis proteins had a dN/dS estimate >1 as calculated for 1-to-1 orthologues between human and mouse, indicating that purifying selection prevailed in the evolution of our dataset. This conclusion is in line with previous studies demonstrating prevalent sequence conservation of male reproductive proteins in various taxa, e.g., in the *Drosophila* sperm proteome^16^, the murine male reproductive tract^17^, and hominoid seminal proteins^29^. But as dN/dS >1 measured over the entire length of a gene is a very conservative threshold of positive selection^30^, it is probable that some of the proteins in our sample contain positions subject to adaptive evolution, although their pairwise dN/dS value between human and mouse was below one (see also ref. 31).

### Suitability of the mouse model in present analyses

The approach to assess survival essentiality of human genes via murine phenotypic KO data has been widely used (see, e.g. refs 32, 33, 34). Nonetheless, phenotypic consequences of gene loss may vary between mouse and human^35^, which might also apply to an unknown number of genes in our dataset. However, Kim *et al*.^36^ reported that proteins essential in yeast, but not mouse nevertheless engage in significantly more interactions in the murine PPI network than proteins which are nonessential in both species. The same authors inferred dN/dS estimates across four yeast species: dN/dS estimates of genes with such differential essentiality status in mouse and yeast closely resembled those of proteins indispensable in both taxa^36^. These similarities have been observed in phylogenetically distant models like mouse and yeast and should hence be even more valid for more closely related taxa such as human and mouse. Therefore, even if some proteins categorized as lethal herein are essential for murine, but not human viability, their node degrees and evolutionary rates should approximate those of proteins essential for survival in both species; a similar pattern should apply to male sub-/infertility proteins.

Furthermore, there are several examples for the transferability of gene essentiality between mouse and human within our dataset. For instance, null mutations in *EIF2AK3, DLD*, or *PDHA1* may result in prepubertal death in both humans and mice (see dataset S1 of ref. 35; and see, e.g., refs 37, 38, 39; for human, see Phenotype MIM numbers 246900, 226980, 312170). Deletion of *Sycp3*, a member of our male sub-/infertility category, causes azoospermia in mice^40^. In humans, lack of testicular *SYCP3* mRNA expression has been implicated in male infertility^41^ and truncating mutations in this gene have been reported in azoospermic patients^42^. Another example from our male infertility category is *CREM*: Ablation of this gene in mice leads to male sterility^43^,^44^,^45^ and altered expression of mRNA or the encoded protein in human spermatids is suspected to underlie some cases of male infertility^46^,^47^.

### Correlates of evolutionary rates in human testis proteins

Spearman’s rank correlations indicated significant interrelations among a protein’s dN/dS, node degree, level of tissue specificity (τ) and multifunctionality in the complete dataset comprising 965 testis proteins (Table 1).

Due to the strong interrelatedness of the incorporated variables, we assumed that the significant correlations between dN/dS and each of the remaining three properties might at least to some extent reflect effects of the other considered variables. In order to disentangle the specific role of single variables in sequence evolution, we employed partial rank correlations between dN/dS and each of the remainder three variables, controlling for the two other properties. This approach revealed that only node degree had a significant partial correlation with dN/dS (Table 2). Hence, the seemingly considerable influence of multifunctionality and tissue specificity on dN/dS values actually reflected variance they shared with node degree. Yet, this does not mean that a gene’s dN/dS is completely independent of its tissue specificity and the number of biological processes in which the encoded protein is involved in. Potentially, within our dataset higher multifunctionality and broader expression entail higher numbers of PPIs, thereby restraining evolutionary rates. This possibility is further addressed in the following sections. Importantly, results of zero-order and partial rank correlations including node degree or τ could be reproduced using alternative approaches to infer these variables (see Materials and Methods, Supplementary Tables S2 and S3).

Negative correlations of evolutionary rates with number of PPIs as described herein have been reported previously for other protein samples and species (see, e.g., refs 26, 48 and 49). Apparently, a major factor underlying this correlation is that highly connected proteins contain a higher proportion of sections with interaction-related functions, each evolving under constraints^26^. In addition, essentiality itself might promote sequence conservation of proteins with high node degree (see, e.g., refs 4, 50 and 51; see also ref. 52). However, Hahn and Kern^48^ demonstrated on the basis of three eukaryotic PPI networks that the interrelation of evolutionary rates and network centrality, as quantified by betweenness centrality, cannot solely be ascribed to a protein’s essentiality status. Following Promislow^53^, they instead hypothesized that proteins more central to interaction networks might be more pleiotropic in terms of functional diversity^53^ and thus more evolutionarily constrained^48^. Such negative relationship between levels of multifunctionality and evolutionary rates, which we also found in zero-order correlations (Table 1), has been attributed to the deleterious effects which substitutions of multifunctional proteins may have on some of the processes they are involved in, even if they are beneficial to others^21^,^54^. As multifunctional proteins are thought to perform their different functions by interaction with varying partners^55^, the effect of multifunctionality on sequence evolution could be partly mediated via numbers of PPIs in the present dataset. Likewise, tissue specificity (τ) may be indirectly linked to sequence evolution via its association with network connectivity. In support of such possibility, broadly expressed proteins are commonly thought to form a core of interactomes, to which tissue-specific proteins are attached as peripheral components, modulating the basal processes carried out by ‘hub’ proteins in a compartment-specific manner^56^,^57^. Although we cannot definitely rule out that zero-order correlations of tissue specificity and multifunctionality with dN/dS were largely spurious, we assume indirect associations of those two variables with sequence evolution (see Table 2). In order to account for such potential indirect effects we kept multifunctionality and tissue specificity in subsequent comparisons among testis protein categories. Nonetheless, it should be emphasized that node degree clearly had the strongest independent link to dN/dS in our testis dataset, as demonstrated by partial rank correlations (Table 2).

### Differences in evolutionary rates, network connectivity, multifunctionality, and tissue specificity among our three protein groups categorized according to their essentiality

We aimed to identify differences among the three categories – male sub-/infertility, lethality, and nonessential proteins – regarding evolutionary rates and with respect to several pleiotropy measures potentially associated with their sequence evolution either directly or indirectly (see above).

Kruskal-Wallis tests revealed highly significant differences among the three categories in their dN/dS, dN, dS, node degree, tissue specificity (τ), and multifunctionality (Table 3). *Post-hoc* Mann-Whitney U (MWU) tests enabled us to determine which categories differed significantly regarding each variable (see below). All presented results which were based on analyses including τ or node degree were recalculated using the same variables inferred with alternative approaches (see Materials and Methods). Results remained unchanged, emphasizing their validity (see Supplementary Table S4 and Fig. S1).

### Differential selective pressures in three testis protein categories

**Figure 1:**
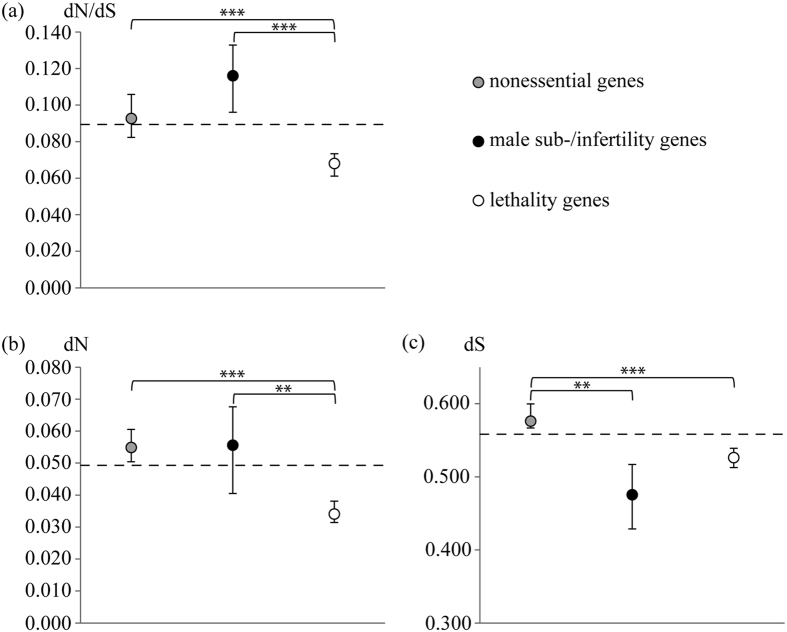
Sequence evolution of three human testis gene groups categorized according to their essentiality. (**a**) Male sub-/infertility genes display highest dN/dS values, followed by the nonessential and the most conserved lethality category. (**b**) Median nonsynonymous substitution rates (dN) of nonessential and male sub-/infertility genes are largely similar, whereas median dN of lethality genes is significantly lower than that of the two other categories. (**c**) While lethality and male sub-/infertility genes are not significantly different regarding their median synonymous substitution rates (dS), median dS of nonessential genes is significantly higher than those of both sub-/infertility and lethality genes. Vertical bars define 95% confidence intervals calculated from 100,000 pseudo-replicates. *** and ** highlight significance at the 0.1% and 1% level, respectively. Significances were corrected against multiple testing using Holm’s procedure (see Materials and Methods). If no asterisk is given, the result of the MWU test is nonsignificant. Dashed lines indicate genome-wide median values of dN/dS, dN, and dS (see Supplementary Materials and Methods).

Our results demonstrate that human testis protein categories associated with various kinds of essentiality evolve under different selective pressures. Gibbs *et al*.^58^ inferred a median K_A_/K_S_ (also: dN/dS) of 0.10 across 11,084 functionally unspecified protein-coding genes, based on 1-to-1 orthologues of human and mouse. While median dN/dS of our nonessential category was only marginally lower than the genome-wide median reported by Gibbs *et al*.^58^, median dN/dS values of lethality and sub-/infertility groups were decreased and increased, respectively (Fig. 1a). Inferring a genome-wide median calculated from 8,610 human protein-coding genes (see Supplementary Materials and Methods; dashed line in Fig. 1a; median dN/dS = 0.089) we were able to reproduce these findings with the exception that nonessential genes showed a median dN/dS value slightly higher than this genome-wide median.

We observed significantly lower dN/dS in the lethality category compared with both nonessential and male sub-/infertility genes; the latter two groups did not differ significantly in their dN/dS (Fig. 1a). Inspection of the underlying numerators and denominators revealed significantly lower dS in genes coding for sub-/infertility than for nonessential proteins, while their median dN values were of about the same level (Fig. 1b and c). Genes associated with male sub- or infertility had significantly higher dN than lethality genes, while the difference regarding dS was nonsignificant; this result corresponds to the findings reported by Torgerson *et al*.^8^. Nonessential genes had both, significantly higher dN and dS than the lethality category (Fig. 1b and c). These results illustrate that highest median dN/dS of sub-/infertility genes was not caused by a generally elevated substitution rate, but rather a decline in dS, although they also displayed significantly higher dN than lethality genes. On the contrary, elevated dN/dS values of genes encoding nonessential proteins compared with lethality genes might partly rely on the generally accelerated substitution rate of the nonessential category. Still, dN apparently increases more than dS in nonessential genes. Finally, lowest dN of lethality genes underscores their strong sequence conservation.

While differences of dN among categories may be attributed to disparity of both mutation rate and selection, variation of dS should theoretically be determined by mutation rates^59^. However, synonymous mutations may also be under selection due to their effects on e.g. mRNA stability^60^, or splicing^61^ as well as translational efficiency (see, e.g., ref. 62). These examples contradict the assumed neutrality of synonymous mutations and argue for nearly neutral evolution of synonymous exchanges (see, e.g. ref. 63). Moreover, dS values may vary in dependence of chromosomal positions: for instance, Torgerson and Singh^64^ reported significantly lower synonymous substitution rates of tissue-specific genes on the X chromosome when compared to those on the autosomes. A similar pattern might partly account for the relatively low median dS of male sub-/infertility genes (Fig. 1c) (see below). Independent of the selective or neutral forces underlying the differences regarding dS among our three testis gene categories, the findings of higher dN in both nonessential and male sub-/infertility genes as compared with the lethality category remain unaffected.

Present evidence for strongest sequence conservation of proteins required for prepubertal survival corresponds to the “knockout-rate prediction”^2^: genes indispensable for viability have been shown to evolve more slowly than genes nonessential in this regard in various taxa, including, e.g., *Escherichia coli*^65^ and mouse^4^. In contrast, protein-coding genes associated with male sub- or infertility displayed highest dN/dS values, an increase that was significant relative to lethality, but not nonessential proteins (Fig. 1a). A trend for accelerated sequence evolution of some male reproductive proteins is a well-documented phenomenon, probably affected by sexual selection (see, e.g., refs 66 and 67).

Several studies unveiled a functional compartmentalization of the sperm proteome: protein-coding genes with functions proximate to fertilization evolve more rapidly than those relevant for more basal steps such as spermatogenesis or sperm assembly^18^,^19^ (see also ref. 16). Based on nucleotide sequences of different mouse strains and species, Vicens *et al*.^18^ found higher proportions of genes with signals of positive selection in groups linked to sperm-egg interaction and sperm motility than in four other categories. They concluded that adaptive evolution of motility-associated proteins could be driven by sperm competition, which might also pertain to a fraction of our sub-/infertility category, such as the proteins encoded by *AKAP4* and *ATP2B4*, which are both linked to (hyperactivated) sperm motility^68^,^69^,^70^,^71^. Vicens *et al*.^18^ moreover identified candidate sites of positive selection in murine *Clgn* whose human orthologue also belongs to our sub-/infertility category. Due to its presumable participation in gamete interaction^72^ (see also ref. 73), coevolution with egg surface proteins could be a factor accelerating its sequence evolution (see, e.g., refs 18 and 74). Hence, coevolutionary processes as well as other forms of postcopulatory sexual selection such as sperm competition might be some of the forces underlying higher median dN/dS values of sub-/infertility genes compared with the two other categories in our dataset and especially in comparison with lethality genes.

### Sequence evolution of immunity and X-chromosomally encoded testis proteins

Since immunity-related proteins (see, e.g., refs 75 and 76) and those encoded on the X chromosome (see, e.g., refs 77 and 78) have been described as rapidly evolving or subject to positive selection, we tested whether such proteins also showed increased rates of sequence evolution in our human testis protein sample. Furthermore, we examined if immunity-related and X-chromosomally encoded proteins were differentially distributed among our three categories, which might have influenced their evolutionary rates. Neither genes coding for proteins involved in immune system processes (GO:0002376; n = 281) nor those encoded on the human X chromosome (n = 40) had significantly higher dN/dS or dN compared with all other members of the dataset (both *p* > 0.05; MWU test). Yet, dS values of immunity-related and X-chromosomal protein-coding genes were significantly higher (*p* < 0.05; MWU test) and lower (*p* < 0.001; MWU test) compared with those of all other genes, respectively. The finding of lower dS values on the X chromosome compared with autosomes corresponds to prior literature (see above and, e.g., ref. 64). Although a smaller proportion of sub-/infertility than lethality or nonessential proteins were involved in immune system processes, the differences among the categories were nonsignificant (Table 4). In contrast, compared with the lethality category, significantly higher proportions of nonessential (*post-hoc* Chi^2^, *p* < 0.001 after correction against multiple testing) and male sub-/infertility proteins (*post-hoc* Fisher’s exact, *p* < 0.05 after correction against multiple testing) were encoded on the human X chromosome (see also Table 4). The difference between nonessential and sub-/infertility proteins regarding their percentage of X-chromosomal genes was nonsignificant (*post-hoc* Fisher’s exact, *p* > 0.05 after correction against multiple testing). We conclude that the different composition of the three protein categories regarding X-encoded and, less so, immunity-related members could have influenced their evolutionary rates, especially their dS values. This might in particular apply to the sub-/infertility category with its low dS and relatively high and low proportions of X-chromosomal and immunity-related proteins, respectively. However, whether or not the varying fractions of proteins participating in immune system processes or encoded on the human X chromosome actually explain some differences among the categories, their influence on the obtained results should only be marginal. In particular, these differences cannot account for the higher median dN/dS in male sub-/infertility genes relative to the nonessential category.

### dN/dS values of our three testis protein categories before the background of node degree, multifunctionality, and tissue specificity

**Figure 2:**
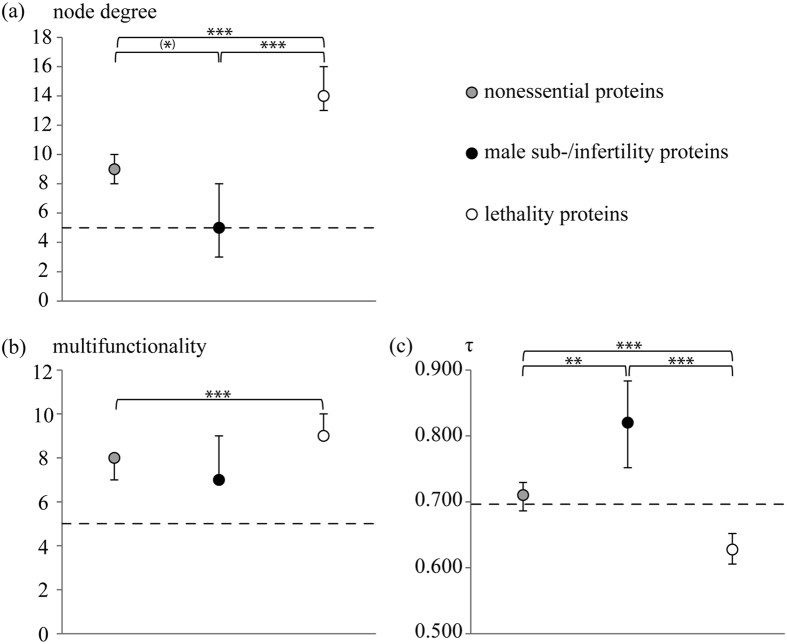
Evolutionary constraints measured as node degree, multifunctionality, and tissue specificity (τ) among three human testis protein groups categorized according to their essentiality. (**a**) Median node degree of lethality proteins is significantly higher than that of male sub-/infertility and nonessential proteins. Nonessential proteins have more PPI partners than male sub-/infertility proteins, but significance is lost after correction against multiple testing (see Materials and Methods). (**b**) Human testis proteins potentially associated with prepubertal lethality are more multifunctional than nonessential and male sub-/infertility proteins. However, only the MWU test contrasting lethality and nonessential proteins gives a significant result. (**c**) Among our three human testis protein categories, male sub-/infertility proteins are most tissue-specific in their expression. Vertical bars define 95% confidence intervals calculated from 100,000 pseudo-replicates. ***, **, and * highlight significance at the 0.1%, 1%, and 5% level, respectively. Significances are corrected against multiple testing using Holm’s procedure (see Materials and Methods). The asterisk in parentheses indicates significance lost after correction against multiple testing. If no asterisk is given, the result of the MWU test is nonsignificant. Dashed lines indicate genome-wide median values of node degree, multifunctionality, and τ (see Supplementary Materials and Methods).

In order to unravel the detailed driving forces behind the differential evolutionary rates among our three testis protein categories we employed measures of evolutionary constraints, i.e., node degree, multifunctionality, and tissue specificity. This approach revealed that proteins in the least divergent lethality category (in terms of dN/dS and dN; see Fig. 1a and b) had significantly higher node degree and were involved in more biological processes than the remaining two groups, although the latter relation was significant only in comparison to nonessential proteins (Fig. 2a and b). Additionally, lethality proteins showed significantly less tissue-biased expression than both, sub-/infertility and nonessential proteins (Fig. 2c). Thus, the lethality category reflected the findings of rank correlations carried out on our entire testis protein sample, whereupon evolutionary conservation combines especially with increased node degree, but also with high multifunctionality, and broad expression (see above). Lethality proteins hence evolve under the influence of constraints imposed by high network connectivity, as well as engagement in a multitude of biological processes and expression in a wide range of tissues. In addition, their indispensability for organismal survival probably further increases the extent of purifying selection operating on their sequences, as outlined above. Therefore, sequence evolution of proteins in the lethality category is constrained by both factors proposed by Wilson *et al*.^1^, namely functional importance and evolutionary (or functional) constraint (see also ref. 59).

In contrast, sequence conservation due to indispensability should not have played a major role in the evolution of nonessential proteins. Accordingly, the median dN/dS value of nonessential genes was significantly higher than that of the lethality category (Fig. 1a), corresponding to findings by other authors (see, e.g. ref. 4; see also ref. 33). Moreover, levels of all measures of evolutionary constraints differed significantly from those in the lethality group (see Fig. 2). Thus, in addition to their higher dispensability, lower levels of evolutionary constraints might have further reduced purifying selection in sequence evolution of nonessential proteins.

Median node degree and numbers of biological processes per protein of the (in terms of dN/dS) most rapidly evolving male sub-/infertility category lay below the respective levels in lethality and nonessential proteins, though only the difference regarding node degree in comparison with lethality proteins was significant after correction against multiple testing (Fig. 2a and b). But with respect to their higher tissue specificity, male sub-/infertility proteins differed significantly from both lethality and nonessential categories (Fig. 2c), reflecting a well-known phenomenon of higher evolutionary rates in proteins expressed with greater tissue bias (see, e.g. refs 3,79 and 80). Higher dispensability, however, should not have impacted the evolution of male sub-/infertility compared to lethality proteins, since both categories are expected to be equally important for an individual’s fitness^8^. Instead, the above findings suggest that relaxation of constraints contributed to the accelerated evolution of male sub- or infertility proteins. Kim *et al*.^27^ described a preferential occurrence of adaptation in noncentral nodes of the human PPI network. One of the explanations discussed by the authors was that more peripheral proteins were less structurally constrained than more central proteins, which might make the former more susceptible to positive selection^27^. A similar pattern might apply to our category of male sub-/infertility proteins since they occupy rather peripheral positions in the human PPI network used herein as evidenced by overall low node degree (Fig. 2a). Notably, their node degree was of the same level as the genome-wide median (see Supplementary Materials and Methods) while the median node degree of the other two groups was higher (Fig. 2a). Moreover, due to their higher tissue specificity compared with the two remaining categories and the genome-wide median (Fig. 2c) male sub-/infertility proteins probably tend to engage in more tissue- and especially testis-specific functions. In combination with their relaxed evolutionary constraints, such testis- or even sperm-specific functions might render sub-/infertility proteins prone to the impact of positive, possibly sexual selection, as described above (see also ref. 8).

In summary, indispensability and evolutionary constraints largely restrain sequence evolution of human testis proteins potentially associated with prepubertal lethality. Nonessential testis proteins are significantly less constrained and should be widely unaffected by functional importance, which both may increase their evolutionary rates. Finally, highest median dN/dS values of proteins linked to male sub- or infertility can be ascribed to the low levels of constraints they evolve under. This relative relaxation of evolutionary constraints and the potential involvement in reproductive functions of some members of the male sub-/infertility category might facilitate adaptive changes in response to postmating sexual selection.

## Conclusion

We showed that essentiality has a major impact on the evolution of human testis proteins. It became evident that proteins associated with male sub- or infertility and those potentially related to the risk of prepubertal death display different patterns regarding their evolutionary conservation, network connectivity, multifunctionality, and tissue-specificity. While proteins linked to prepubertal death were strongly conserved, more widely expressed, highly connected and multifunctional, the category associated with male sub- or infertility evolved more rapidly, had strongly tissue-biased expression, and exhibited low connectivity and relatively reduced numbers of biological processes. These results suggest that among the three categories studied, constraints are strongest in lethality proteins, while most relaxed in male sub-/infertility proteins. Accelerated evolution of the latter protein category might, moreover, partly be ascribed to impact of sexual selection.

Noteworthy, we used a rather conservative approach by including only proteins whose expression in human testis was reported to be supportive in the Human Protein Atlas (see Materials and Methods). Furthermore, interactomes relying on our present knowledge about PPIs are largely incomplete^81^. Thus, some peripheral proteins might have been left unconsidered in the present study. Additionally, it is possible that some proteins herein categorized as nonessential or lethal are associated with female sub- or infertility. Such proteins are expected to evolve at similar rates as male sub- or infertility proteins^8^ and might consequently slightly have blurred our results. The fact that we detected distinct patterns in our dataset despite the potential limitations of our approach evidences the strength of the described relationships.

In spite of the opposing evolutionary forces reported herein, several researchers combined proteins indispensable for survival and those required for reproduction into one single essential category (see, e.g., refs 3 and 36). However, according to our results it is advisable to differentiate between sub-/infertility and lethality proteins in future investigations on the evolution of essential proteins.

Finally, our observations demonstrate that PPI network connectivity and dN/dS values may be useful tools to identify proteins essential for male fertility. Also tissue specificity and less so multifunctionality inform about the essentiality status of testis proteins but these measures alone are insufficient to discriminate differential essentiality statuses in testis proteins. This knowledge is relevant since infertility affects 10–15% of couples worldwide (see, e.g., refs 82 and 83). Also, proteins with testis-specific expression and function might be prime targets for male non-hormonal contraception, since side effects in other tissues than testis can largely be excluded.

## Materials and Methods

### Dataset of proteins from human seminiferous tubules

Proteins expressed in the seminiferous ducts of human testis under physiological conditions were extracted from “normal tissue” data of the Human Protein Atlas^84^ (http://www.proteinatlas.org/) version 12. We chose proteins from seminiferous tubules for our analyses as they constitute a large part of testes and are the location of spermatogenesis and spermiogenesis. For reasons of simplicity, we call the members of our dataset “testis proteins”, though we are aware that testes consist of more constituents. In order to ensure high quality of the data, we exclusively considered 2,986 proteins whose expression in seminiferous ducts was designated “supportive” in the Human Protein Atlas data. Most Ensembl IDs (version 73) provided in the respective downloadable “normal tissue” data were also available in Ensembl version 82, which we used for subsequent analyses (see below). Altogether 53 genes whose IDs were unavailable in Ensembl version 82 due to differences between the two genome assemblies were mapped to new IDs either via Ensembl Biomart or manually using their gene names or the Uniprot IDs specified in Ensembl version 73. To match the respective proteins to Human Protein Atlas RNA sequencing data and expression values provided in the supplementary data of Kryuchkova-Mostacci and Robinson-Rechavi^85^, we used their old IDs (Ensembl version 73; see below). For each protein we determined the human chromosome on which the corresponding gene is encoded using Ensembl Biomart (version 82) to distinguish between X-chromosomal and all other genes.

### Human protein-protein interaction (PPI) network

Node degree values for each protein were extracted from a human PPI network. We used the network published as supplementary data of the article by Chapple *et al*.^55^, comprising only experimentally verified binary interactions, as a starting point for our network construction. The interactions represented by this network were not confined to testes, but rather combined PPIs from diverse tissues. The Uniprot ID mapping tool was employed to map the entry names to current Uniprot accession numbers (state: November 2015). Proteins which had been deleted from Uniprot since the study by Chapple *et al*.^55^ were removed from the network. If a Uniprot entry name from the dataset by Chapple *et al*.^55^ had been mapped to another entry name, the old was replaced by the new one. Subsequently, we extracted corresponding Ensembl Gene IDs for each protein, again using the Uniprot ID mapping tool. If this procedure failed, we obtained their Ensembl Gene IDs via Ensembl Biomart version 82 using their Swiss-Prot/TrEMBL accession numbers or their associated gene names if provided. In some cases, gene names first had to be assigned to their current gene symbol or its synonyms via HGNC (HUGO Gene Nomenclature Committee; http://www.genenames.org/). As we aimed to solely include functional PPIs, we ignored all proteins whose genes represented a biotype other than “protein_coding” according to Ensembl. Thus, we excluded, inter alia, pseudogenes, antisense-genes, but also immunoglobulin und T-cell receptor genes as they constitute gene segments rather than protein-coding genes in a strict sense. Proteins with given gene names which could not be related to their Ensembl Gene IDs or were denoted not to be protein-coding in Ensembl were left in the network if they were a “protein-coding gene” according to HGNC. If one Uniprot accession number had several Ensembl Gene IDs on the primary assembly of the human genome, it was also left in the network, but its Ensembl IDs were ignored in further analyses; if it had several Ensembl Gene IDs, but only one on the primary assembly, all others which were not assigned to the primary assembly were discarded. Two further proteins (LC7L2_HUMAN, STBD1_HUMAN) were kept without Ensembl Gene IDs because they had been assigned to readthrough proteins via Ensembl Biomart version 82. Uniprot accession numbers which could not be matched to Ensembl Gene IDs, had no given or identifiable gene name, or were not coding for a functional protein were deleted from the network. If one or more TrEMBL IDs corresponded to an Ensembl Gene ID which was also assigned to a Swiss-Prot ID, the TrEMBL accession numbers were deleted and their interactions inherited by the Swiss-Prot protein. However, mapping of one Ensembl Gene ID to two or more Swiss-Prot accession numbers was accepted (see below). Additionally, since the compilation of the network data by Chapple *et al*.^55^ the Swiss-Prot entry names CSH_HUMAN and HSP71_HUMAN each had been demerged into two proteins. As neither of these proteins was contained in our dataset of human testis proteins, we left them in the network as single proteins with their original Uniprot entry names, but without related Ensembl IDs. The final network comprised 12,144 nodes (proteins) and 71,765 edges (interactions).

Although the analyses presented within this article are based on the human PPI network which was built as described above, we analyzed two additional interactomes, for which results are given in the supplementary data. In one alternative network, CSH_HUMAN and HSP71_HUMAN were split into the proteins CSH1_HUMAN and CSH2_HUMAN as well as HS71A_HUMAN and HS71B_HUMAN, respectively, each adopting all interactions of the original protein. Thereby, B1A4H9_HUMAN, which represented the TrEMBL entry version of CSH2, was deleted from the network and its interactions were transferred to the Swiss-Prot entry CSH2_HUMAN (overall: 12,145 nodes, 71,842 edges). The second alternative network was an adapted version of the original interactome by Chapple *et al*.^55^, with only the deletions and changes of Uniprot accession numbers since its publication incorporated and each, CSH_HUMAN and HSP71_HUMAN, kept as single protein (overall: 12,595 nodes, 73,367 edges).

We employed Cytoscape^86^ version 2.8.3 to edit the network and remove duplicated edges as well as self-interactions and used the plugin NetworkAnalyzer^87^ to extract node degree values. Ensembl Gene IDs corresponding to proteins expressed in human seminiferous ducts according to Human Protein Atlas version 12 (see above) were mapped to the Ensembl Gene IDs representing the Uniprot IDs from our PPI network. If an Ensembl Gene ID was linked to more than one Swiss-Prot ID in the network, we chose the one with highest node degree for analysis and for extraction of gene ontology (GO) annotations (see below); if an Ensembl Gene ID corresponding to a protein from our testis dataset was not found to be linked to any Uniprot ID in the network, we excluded it from further analyses.

### Identifying potential associations of proteins with human lethality and male sub- or infertility

The use of targeted KO mutants for assessing gene essentiality has been applied before (see, e.g., refs 34 and 52). We identified murine 1-to-1 orthologues of the human genes incorporated in this study and subsequently matched them with their MGI (Mouse Genome Informatics) IDs via Ensembl Biomart (version 82). All information concerning mouse KOs used in this study relied on files downloaded from MGI^88^ (http://www.informatics.jax.org/) on August 19^th^ 2015 (MGI version 5.22).

Known phenotypic alleles generated by homologous recombination (allele type “Targeted”) were extracted from the file MGI_PhenotypicAllele.rpt. We exclusively considered alleles whose allele attributes contained the term “Null/knockout” and which affected single genes. To ensure the latter, we solely included alleles with a single MGI Marker Accession ID in MGI_PhenoGenoMP.rpt. Furthermore, if more than one gene name was specified in an allele symbol (as indicated by a forward slash) we checked if in fact more than one gene was targeted and excluded alleles from categorization in such cases. To classify genes we accepted homozygous or – in case of X-chromosomal genes in male mice – hemizygous targeted null mutations.

If an MP (Mammalian Phenotype) ID was used for classification with all its subterms, we downloaded these subterm IDs from MouseMine^89^ (state: September 2015; http://www.mousemine.org/mousemine/begin.do). Single MP IDs were extracted from the Mammalian Phenotype Ontology at MGI (http://www.informatics.jax.org/searches/MP_form.shtml). A list of all MP IDs used is provided in the supplementary data (Tables S5 and S6).

Lethality genes were associated with any of the MP IDs subsumed under “preweaning lethality” (MP:0010770), or “lethality at weaning” (MP:0008569). These MP IDs are assigned to alleles which decrease viability so that lower than Mendelian ratios of individuals with homo- or hemizygous null mutations appear at some time point from their prenatal period up to three to four weeks of age. They are thus linked to death before puberty, which initiates in slightly older mice (see http://www.informatics.jax.org/mgihome/other/mouse_facts1.shtml^90^). Genes with alleles designated with “complete lethality” (MP:0011400), “partial lethality” (MP:0010831), “decreased survivor rate” (MP:0008770), or “abnormal survival” (MP:0010769) – all as single MP IDs without subterms – were integrated into the lethality category if these null mutations resulted in death of some or all individuals aged up to four weeks leading to the appearance of lower than Mendelian ratios, as derived from the literature cited in MGI_PhenoGenoMP.rpt. The selection of lethality phenotypes (see supplementary data, Table S5) agreed largely with that in ref. 4.

If the homo- or hemizygous targeted KO of a gene resulted in male in- or subfertility (MP:0001925 or MP:0001922), it was categorized as male sub-/infertility gene. Thereby, we accepted all causes of male in-/subfertility (see below). If quoted, these alleles were checked in the original literature specified in MGI_PhenoGenoMP.rpt. Genes for which targeted null mutations were reported to cause other male reproductive abnormalities – for instance in spermatogenesis, male reproductive system morphology or physiology – were also included in the group of male sub-/infertility genes if inspection of the literature given in MGI files confirmed male sub-/infertility or noticeable reduction of male fertility in terms of pregnancy frequencies (for all MP IDs used, see supplementary data, Table S6). For instance, this was the case in mice with arrest of male meiosis before its completion (e.g., *Brdt*^91^) or sex reversal (e.g., *Ar*, see, e.g., ref. 92). Moreover, the human orthologue of the protein encoded by murine *Zbtb16* was included in the male sub-/infertility group although male KO mice were neither azoospermic nor described as infertile; yet, Costoya *et al*.^93^ stated that the low numbers of viable sperm from these mice made *in vitro* fertilization impossible, indicating serious fertility dysfunction. Applying the outlined criteria, KOs of murine orthologues of most proteins in the male sub-/infertility category led to sterility or fertility issues due to defects manifesting in testis or sperm. However, two members of this category were linked to male sub- or infertility in mice due to erectile (*Stam*^94^) or ejaculatory (*Etv4* or *Pea3*^95^) dysfunction. The latter protein was also part of the fertility sample studied by Torgerson *et al*.^8^. Despite different underlying causes of male reproductive disturbances, all members of the male sub-/infertility category were essential for full fertility in male mice. If the available null mutations of a gene were exclusively associated with fertility issues via “decreased litter size” (MP:0001935) and this phenotype occurred in homozygous couples or could possibly be attributed to male KO mutants, the gene was excluded from further analyses due to the problematical transferability of this phenotype to human reproduction. Generally, we removed genes from the dataset if their ablation only resulted in reproductive abnormalities in homozygous couples, while mice were basically fertile. Moreover, genes were eliminated from the dataset if they were associated with any of the male reproduction-related phenotype IDs used (see Supplementary Table S6), but males were described as fertile in the quoted literature or the given information were insufficient to evaluate fertility. Thereby, we also excluded genes associated with phenotypes potentially increasing fertility, such as elevated sperm counts or enlarged testes, to avoid that the nonessential or lethality (see below) categories contain genes linked to any male reproductive abnormalities. We furthermore left genes with KO phenotypes only emerging upon manipulation of additional factors such as food composition out of the final dataset. Three more proteins were removed due to the following reasons: First, *NRIP1* was excluded since male mice with a null mutation of this gene were fertile, but produced fewer homozygous offspring than expected, which questions the viability assumed for these null mutants^96^. Second, Cesari *et al*.^97^ reported that the infertility of hemizygous male *Elk1*-KO mice was probably due to aberrant expression of the *HygTk* fusion gene which had been used to replace the coding sequence. Thus, we left ELK1 unconsidered in statistical analyses. Third, male mice lacking functional Rad18 protein initially exhibited normal fertility comparable to wild-type littermates, which, however, was reduced at 12 months of age^98^. Although fertility issues in men with advanced age are indeed an important factor in andrology (see, e.g., ref. 99), we removed RAD18 from our dataset because mice younger than 12 months appeared fertile. If a gene was connected to MP IDs potentially applying to both sexes (such as the single MP ID “infertility” (MP:0001924)), but was found to be related to female-specific reproductive phenotypes in the cited articles, it was classified as non-associated with male sub- or infertility, thus being nonessential or lethal (see below). The same applies to female-specific subterms of “abnormal sex determination” (MP:0002210; see also Supplementary Table S6).

Finally, we excluded genes from analyses which were related to both prepubertal death and male reproductive anomalies, also if these were evoked by different alleles. Thereby, any of the reproductive phenotypes listed in Supplementary Table S6 including “decreased litter size” was taken into account, unless they exclusively described female abnormalities (see above). For one gene excluded due to such double essentiality, *XRCC5*, description of reduced litter size was not found in the article quoted in MGI files^100^, but was instead confirmed via the original publication^101^, which was cited in Henrie *et al*.^100^.

All protein-coding genes for which phenotypic homo- or hemizygous KO alleles were available in the used MGI data, but which were neither associated with prepubertal lethality nor with male sub- or infertility as described above and which were not excluded from the dataset due to the aforementioned reasons, were categorized as nonessential.

### Assessing selection and tissue specificity

In order to determine the extent and direction of selection acting on each protein, we extracted dN/dS estimates from ENSEMBL version 82, which had been calculated using CodeML as implemented in the PAML package^102^. We exclusively collected dN/dS values derived from 1-to-1 orthologues of human (*Homo sapiens*; genome assembly GRCh38.p3) and mouse (*Mus musculus*; genome assembly GRCm38.p4). Since we considered only dN/dS estimates reported on the orthologues view pages of the genes included, we avoided values potentially biased by saturation of dS, which are masked out on these pages (http://sep2015.archive.ensembl.org/info/genome/compara/homology_method.html). For all proteins for which dN/dS values could be extracted from their Ensembl pages, we additionally derived dN and dS values via Ensembl Biomart (version 82).

We investigated expression specificity of each protein using the tissue specificity index τ. Values of τ vary between 0 for genes expressed at similar levels in all examined tissues and 1 for genes expressed in only one tissue^20^. Following Kaiser *et al*.^103^ τ was based on FPKM (fragments per kilobase of transcript per million fragments mapped) values which we downloaded from Human Protein Atlas version 12^104^. Excluding data referring to three female-specific tissues (ovary, placenta, and uterus), we used FPKM values from altogether 24 tissues to calculate τ. In supplementary data (Tables S2–S4, Fig. S1), we also report analyses including this variable in which τ is based on all 27 tissues from Human Protein Atlas version 12 and with τ extracted from supplementary data by Kryuchkova-Mostacci and Robinson-Rechavi^85^, which was calculated with the same data, but different methodology. In analyses including the latter values of τ, one gene *RECQL4*, a member of the lethality category, was missing from the dataset since its tissue specificity index had not been calculated by Kryuchkova-Mostacci and Robinson-Rechavi^85^.

### Gene ontology (GO) categorization

The level of multifunctionality was defined as the number of biological processes in which a protein is involved in^21^. To infer protein-specific values, we mapped biological process annotations from the human GOA (Gene Ontology Annotation) file (state: November 9^th^ 2015; http://www.ebi.ac.uk/GOA/downloads) to GOSlim generic terms using map2slim. Numbers of nonredundant biological processes per protein were counted, thereby ignoring the term “biological process” (GO:0008150) if combined with the evidence code “ND”, as it indicates unavailability of information (https://www.ebi.ac.uk/QuickGO/GTerm?id=GO:0008150). Proteins involved in immune system processes (GO:0002376) were also identified from these GOSlim annotations.

### Statistical analyses

All statistical analyses were conducted in SPSS version 22 (IBM) unless stated otherwise. Analyses were based exclusively on proteins with all variables available and assigned to one of three categories according to their essentiality, i.e. lethality, male sub-/infertility, or nonessential proteins (see above). Overall, 965 proteins were considered. We performed Spearman’s rank correlations (two-tailed) between each possible pair of the following four variables to study potential relationships among them: dN/dS, node degree, multifunctionality, and tissue specificity (τ). In order to further disentangle the contributory role of single variables in sequence evolution of the sampled proteins, we conducted pairwise partial rank correlations (two-tailed) between dN/dS estimates and the three other variables in Matlab R2015b, controlling for the remaining two variables. Potential differences regarding the mentioned four variables as well as dN and dS were investigated among the three protein categories applying Kruskal-Wallis tests. We conducted *post-hoc* MWU tests (two-tailed) between each pair of protein categories for each variable. Furthermore, MWU tests were employed to compare dN/dS, dN, and dS values of X-chromosomal or immunity-related proteins with all other members of the dataset in each case. Using Chi^2^ tests (two-tailed) we tested for differential distribution of X-chromosomal or immunity proteins among our three testis protein categories. If the result of the Chi^2^ test was significant, we conducted pairwise *post-hoc* Chi^2^ tests (two-tailed) between each pair of the three testis protein categories. Fisher’s exact test was utilized if the expected values in any of the cells of the contingency table were below 5. To account for problems of multiplicity in *post-hoc* tests we applied Holm’s procedure. Furthermore, *p*-values of correlations including dN/dS were calculated for both zero-order and partial correlations and were thus also adjusted with Holm’s procedure. Finally, all analyses including tissue specificity (τ) and node degree were conducted repeatedly because we additionally calculated these variables by alternative approaches (see above). Therefore, we adjusted their *p*-values according to the total number of similar tests performed, also taking into account the aforementioned multiplicity issues. 95% confidence intervals of medians were calculated with a bootstrap algorithm building 100,000 pseudo-replicates.

## Additional Information

**How to cite this article:** Schumacher, J. *et al*. Effects of different kinds of essentiality on sequence evolution of human testis proteins. *Sci. Rep.* **7**, 43534; doi: 10.1038/srep43534 (2017).

**Publisher's note:** Springer Nature remains neutral with regard to jurisdictional claims in published maps and institutional affiliations.

## Supplementary Material

Supplementary Figure S1

Supplementary Table S1

## Figures and Tables

**Table 1:** Results of Spearman’s rank correlations between studied variables.

Correlation between	Spearman’s correlation coefficient; *p*^a^
dN/dS, node degree	ρ = −0.229***
dN/dS, multifunctionality	ρ = −0.134***
dN/dS, τ	ρ = 0.088*(*)
node degree, multifunctionality	ρ = 0.398***
node degree, τ	ρ = −0.304***
τ, multifunctionality	ρ = −0.082*

^a^All *p*-values were adjusted with Holm’s procedure (see Materials and Methods). ***, **, and * highlight significance at the 0.1%, 1%, and 5% level, respectively. The asterisk in parentheses indicates significance lost after correction against multiple testing.

**Table 2:** Results of partial rank correlations between dN/dS estimates and three other variables.

Correlation between | controlling for	Spearman’s partial correlation coefficient; *p*^a^
dN/dS, node degree | multifunctionality, τ	ρ = −0.178***
dN/dS, multifunctionality | node degree, τ	ρ = −0.049^ns^
dN/dS, τ | node degree, multifunctionality	ρ = 0.022^ns^

^a^All *p*-values were adjusted with Holm’s procedure (see Materials and Methods). ***Highlight significance at the 0.1% level; ns, nonsignificant.

**Table 3:** Results of Kruskal-Wallis tests among three protein categories for six variables.

Variable	*H*	*p*^a^
dN/dS	43.334	***
dN	54.360	***
dS	29.781	***
node degree	45.898	***
τ	60.307	***
multifunctionality	26.273	***

^a^For node degree and τ, *p*-values were adjusted with Holm’s procedure (see Materials and Methods). ***highlight significance at the 0.1% level.

**Table 4:** Proportion of proteins in three human testis protein categories with immunity-related functions or encoded on the X chromosome.

	nonessential	sub-/infertility	lethality	Statistical test	
immunity	32.0% (130/406)	19.3% (11/57)	27.9% (140/502)	4.687^a^	ns
X chromosome	6.7% (27/406)	8.8% (5/57)	1.6% (8/502)		***^b^

Numbers of proteins in each category which are involved in immune system processes or encoded on the human X chromosome and total numbers of proteins per category are given in parentheses; ^a^Pearson’s Chi^2^. ***Highlight significance at the 0.1% level; ns, nonsignificant. ^b^*p* of Fisher’s exact test.
