# Supplementary material for: Effects of different kinds of essentiality on sequence evolution of human testis proteins
Source: Sci Rep. 2017 Mar 8;7:43534. doi: 10.1038/srep43534 (PMC5341092; doi:10.1038/srep43534)
Supplement: Supplementary Figure S1 [file srep43534-s1.doc]

**Supplementary Figure S1, Supplementary Tables S2-S6, and Supplementary Materials and Methods to “Effects of different kinds of essentiality on sequence evolution of human testis proteins” (Julia Schumacher, Hans Zischler, Holger Herlyn)**

**Table S2. Results of Spearman’s rank correlations between studied variables if node degree or tissue specificity (τ) was included.**

| **Correlation between** | **Spearman’s correlation coefficient; *p*^a^** |
| --- | --- |
| dN/dS, node degree^b^ | ρ = -0.229; *** |
| dN/dS, node degree^c^ | ρ = -0.230; *** |
| dN/dS, τ^d^ | ρ = 0.099; ** |
| dN/dS, τ^e^ | ρ = 0.116; **(*) |
| node degree^b^, multifunctionality | ρ = 0.399; *** |
| node degree^c^, multifunctionality | ρ = 0.398; *** |
| τ^d^, multifunctionality | ρ = -0.087; *(*) |
| τ^e^, multifunctionality | ρ = -0.071; * |
| node degree^b^, τ^f^ | ρ = -0.304; *** |
| node degree^c^, τ^f^ | ρ = -0.304; *** |
| node degree^g^, τ^d^ | ρ = -0.319; *** |
| node degree^g^, τ^e^ | ρ = -0.301; *** |

^a^***, **, and * highlight significance at the 0.1 %, 1 %, and 5 % level, respectively. All *p*-values were adjusted with Holm’s procedure. Asterisks in parentheses indicate significances lost after correction against multiple testing. ^b^node degree as calculated in the main text, but based on a network with CSH_HUMAN and HSP71_HUMAN split into two proteins, each (“first alternative network”); ^c^node degree derived from a network with only deletions and changes in Uniprot incorporated which occurred since the network construction by Chapple et al.^1^ (“second alternative network”); ^d^tissue specificity τ calculated based on expression data from 27 human tissues; ^e^tissue specificity τ as extracted from supplemental material by Kryuchkova-Mostacci and Robinson-Rechavi^2^; if this variable is used, 964 instead of 965 proteins are included in analyses (see Materials and Methods section of the main text); ^f^tissue specificity τ as used for analyses presented in the main text; ^g^node degree as used for analyses presented in the main text.

**Table S3. Results of partial Spearman’s rank correlations between dN/dS and node degree or tissue specificity (τ) as calculated by alternative approaches.**

| **Correlation between | controlling for** | **Spearman’s partial correlation coefficient; *p*^a^** |
| --- | --- |
| dN/dS, node degree^b^ | multifunctionality,τ^f^ | ρ = -0.178; *** |
| dN/dS, node degree^c^ | multifunctionality,τ^f^ | ρ = -0.179; *** |
| dN/dS, τ^d^ | multifunctionality,node degree^g^ | ρ = 0.031; ns |
| dN/dS, τ^e^ | multifunctionality,node degree^g^ | ρ = 0.053; ns |

^a^*** highlight significance at the 0.1 % level; ns, nonsignificant. All *p*-values were adjusted with Holm’s procedure. ^b^node degree as calculated in the main text, but based on a network with CSH_HUMAN and HSP71_HUMAN split into two proteins, each (“first alternative network”); ^c^node degree derived from a network with only deletions and changes in Uniprot incorporated which occurred since the network construction by Chapple et al.^1^ (“second alternative network”); ^d^tissue specificity τ calculated based on expression data from 27 human tissues; ^e^tissue specificity τ as extracted from supplemental material by Kryuchkova-Mostacci and Robinson-Rechavi^2^; if this variable is used, 964 instead of 965 proteins are included in analyses (see Materials and Methods section of the main text); ^f^tissue specificity τ as used for analyses presented in the main text; ^g^node degree as used for analyses presented in the main text.

**Table S4. Results of Kruskal-Wallis tests among three protein categories for node degree or tissue specificity (τ) as calculated by alternative approaches.**

| **Variable** | ***H*** | ***p*^a^** |
| --- | --- | --- |
| node degree^b^ | 45.903 | *** |
| node degree^c^ | 45.997 | *** |
| τ^d^ | 63.881 | *** |
| τ^e^ | 74.095 | *** |

^a^*** highlight significance at the 0.1 % level. All *p*-values were adjusted with Holm’s procedure. ^b^node degree as calculated in the main text, but based on a network with CSH_HUMAN and HSP71_HUMAN split into two proteins, each (“first alternative network”); ^c^node degree derived from a network with only deletions and changes in Uniprot incorporated which occurred since the network construction by Chapple et al.^1^ (“second alternative network”); ^d^tissue specificity τ calculated based on expression data from 27 human tissues; ^e^tissue specificity τ as extracted from supplemental material by Kryuchkova-Mostacci and Robinson-Rechavi^2^; if this variable is used, 964 instead of 965 proteins are included in analyses (see Materials and Methods section of the main text).

**
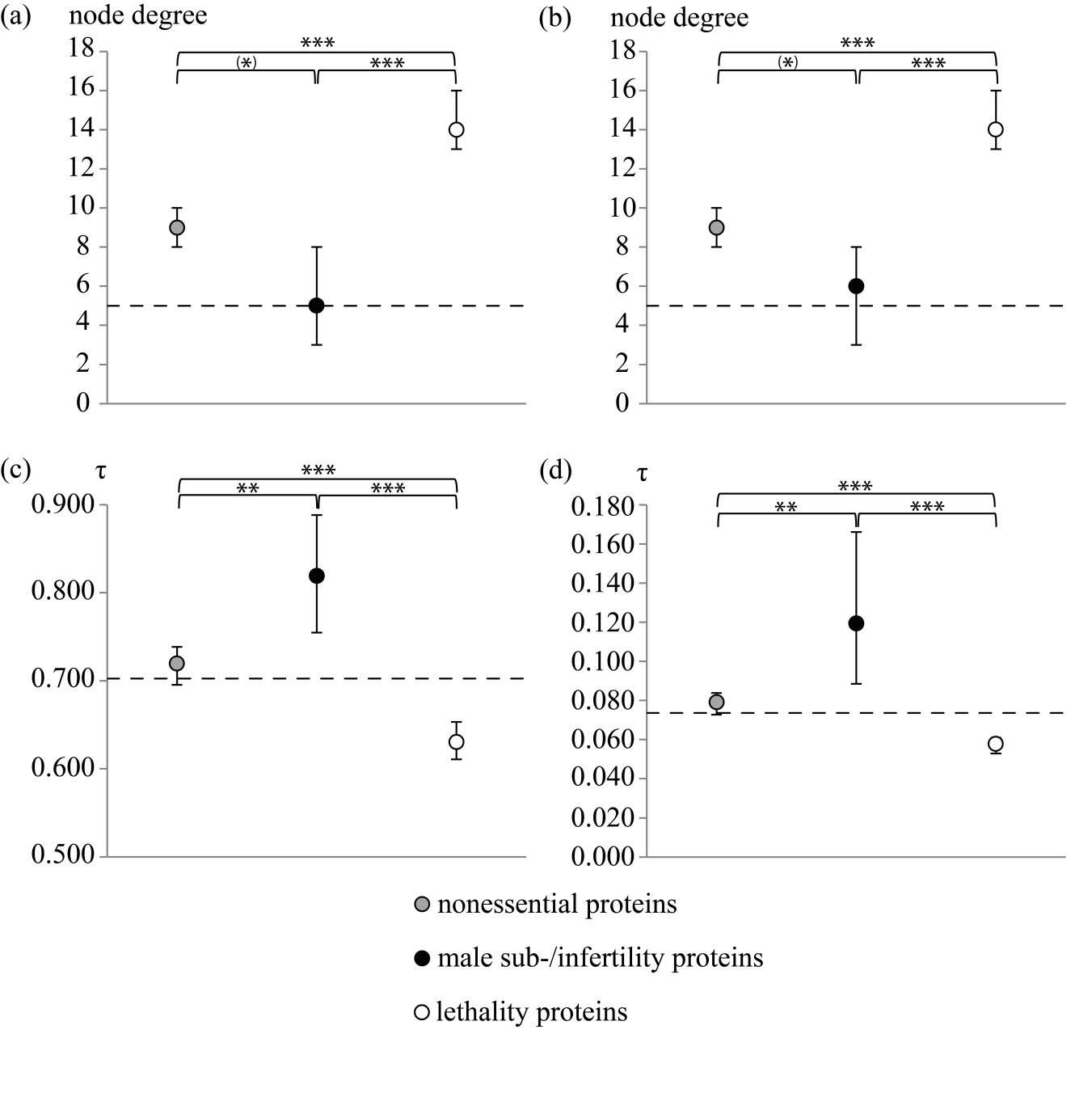
**

**Figure S1.** Evolutionary constraints measured as node degree or tissue specificity (τ), each calculated with two alternative approaches. All results remain unchanged compared to those presented in the main text. (a) Node degree as calculated in the main text, but based on a network with CSH_HUMAN and HSP71_HUMAN split into two proteins, each (“first alternative network”). Nonessential proteins have more PPI partners than male sub-/infertility proteins, but significance is lost after correction against multiple testing. (b) Node degree derived from a network with only deletions and changes in Uniprot incorporated which occurred since the network construction by Chapple et al.^1^ (“second alternative network”). Nonessential proteins have more PPI partners than male sub-/infertility proteins, but significance is lost after correction against multiple testing. (c) τ calculated on the basis of FPKM values for 27 human tissues derived from the Human Protein Atlas version 12. (d) τ as extracted from supplemental material by Kryuchkova-Mostacci and Robinson-Rechavi^2^. 964 instead of 965 proteins are included in analyses when using these τ values (see Materials and Methods section of the main text). Vertical bars define 95 % confidence intervals calculated from 100,000 pseudo-replicates. All *p*-values were adjusted with Holm’s procedure. ***, **, and * highlight significance at the 0.1 %, 1 %, and 5 % level, respectively. Asterisks in parentheses indicate significances lost after correction against multiple testing. If no asterisk is given, the result of the MWU test is nonsignificant. Dashed lines indicate genome-wide median values of node degree and τ (see Supplementary Materials and Methods).

**Supplementary Materials and Methods**

Genome-wide medians for all incorporated variables were calculated from a dataset comprising 8,610 protein-coding genes. To generate the background dataset, we started from all protein-coding genes encoded on human chromosomes and mtDNA as derived from Ensembl Biomart version 82. For these, we extracted dN/dS values which had been calculated based on human and mouse 1-to-1 orthologues from Ensembl version 82 as described in the Materials and Methods section of the main text. For the genes for which dN/dS estimates were available in Ensembl version 82, node degree and number of biological processes (multifunctionality) according to GOSlim were determined, again according to the descriptions in the main text. The procedure to calculate or extract τ from Human Protein Atlas (version 12) data^3^ or from supplementary data by Kryuchkova-Mostacci and Robinson-Rechavi², respectively, also conforms to the approaches described in the main text. However, to gather these expression data, several Ensembl Gene IDs had to be mapped from version 82 to version 74 or 67 (version 73 was not available any more in the Ensembl Archive at the time of data collection); this was achieved by manual mapping of gene names between the different Ensembl versions. Finally, we extracted dN and dS estimates calculated from 1-to-1 orthologues of human and mouse from Ensembl Biomart version 82. To calculate medians of the genome background, which we depict in all figures, we exclusively used genes for which all variables (dN/dS, dN, dS, node degree, τ as calculated based on Human Protein Atlas version 12 data³, and multifunctionality) were available, except for τ gathered from supplementary data by Kryuchkova-Mostacci and Robinson-Rechavi². Median of the latter variable was calculated based on 8,594 instead of 8,610 genes; for the remaining 16 genes, one of which was *RECQL4* (see Materials and Methods of the main text), τ had not been calculated by Kryuchkova-Mostacci and Robinson-Rechavi².

**Table S5**. Phenotype IDs used to assemble proteins associated with prepubertal lethality.

| **MP^a^ ID** | **MP^a^ term** |
| --- | --- |
| MP:0002058 | neonatal lethality |
| MP:0002080 | prenatal lethality |
| MP:0002081 | perinatal lethality |
| MP:0002082 | postnatal lethality |
| MP:0006204 | embryonic lethality before implantation |
| MP:0006205 | embryonic lethality between implantation and somite formation |
| MP:0006206 | embryonic lethality between somite formation and embryo turning |
| MP:0006207 | embryonic lethality during organogenesis |
| MP:0006208 | lethality throughout fetal growth and development |
| MP:0008527 | embryonic lethality at implantation |
| MP:0008762 | embryonic lethality |
| MP:0009850 | embryonic lethality between implantation and placentation |
| MP:0010770 | preweaning lethality |
| MP:0010832 | lethality during fetal growth through weaning |
| MP:0011085 | complete postnatal lethality |
| MP:0011086 | partial postnatal lethality |
| MP:0011087 | complete neonatal lethality |
| MP:0011088 | partial neonatal lethality |
| MP:0011089 | complete perinatal lethality |
| MP:0011090 | partial perinatal lethality |
| MP:0011091 | complete prenatal lethality |
| MP:0011092 | complete embryonic lethality |
| MP:0011093 | complete embryonic lethality at implantation |
| MP:0011094 | complete embryonic lethality before implantation |
| MP:0011095 | complete embryonic lethality between implantation and placentation |
| MP:0011096 | complete embryonic lethality between implantation and somite formation |
| MP:0011097 | complete embryonic lethality between somite formation and embryo turning |
| MP:0011098 | complete embryonic lethality during organogenesis |
| MP:0011099 | complete lethality throughout fetal growth and development |
| MP:0011100 | complete preweaning lethality |
| MP:0011101 | partial prenatal lethality |
| MP:0011102 | partial embryonic lethality |
| MP:0011103 | partial embryonic lethality at implantation |
| MP:0011104 | partial embryonic lethality before implantation |
| MP:0011105 | partial embryonic lethality between implantation and placentation |
| MP:0011106 | partial embryonic lethality between implantation and somite formation |
| MP:0011107 | partial embryonic lethality between somite formation and embryo turning |
| MP:0011108 | partial embryonic lethality during organogenesis |
| MP:0011109 | partial lethality throughout fetal growth and development |
| MP:0011110 | partial preweaning lethality |
| MP:0011111 | complete lethality during fetal growth through weaning |
| MP:0011112 | partial lethality during fetal growth through weaning |
| MP:0013292 | embryonic lethality prior to organogenesis |
| MP:0013293 | embryonic lethality prior to tooth bud stage |
| MP:0013294 | prenatal lethality prior to heart atrial septation |
| MP:0008569 | lethality at weaning |
| MP:0011083 | complete lethality at weaning |
| MP:0011084 | partial lethality at weaning |
| MP:0011400^b^ | complete lethality |
| MP:0010831^b^ | partial lethality |
| MP:0008770^b^ | decreased survivor rate |
| MP:0010769^b^ | abnormal survival |

^a^MP, mammalian phenotype; ^b^alleles associated with these MP IDs were included in the lethality category if they accorded to the specifications described in the Materials and Methods section of the main text.

**Table S6. Phenotype IDs used to assemble proteins associated with male sub- or infertility.**

| **MP^a^ ID** | **MP^a^ term^b^** |
| --- | --- |
| MP:0001925 | male infertility |
| MP:0001922 | reduced male fertility |
| MP:0001155 | arrest of spermatogenesis |
| MP:0001156 | abnormal spermatogenesis |
| MP:0001932 | abnormal spermiogenesis |
| MP:0002685 | abnormal spermatogonia proliferation |
| MP:0002686 | globozoospermia |
| MP:0002687 | oligozoospermia |
| MP:0004182 | abnormal spermiation |
| MP:0005159 | azoospermia |
| MP:0005169 | abnormal male meiosis |
| MP:0005578 | teratozoospermia |
| MP:0006379 | abnormal spermatocyte morphology |
| MP:0006380 | abnormal spermatid morphology |
| MP:0008261 | arrest of male meiosis |
| MP:0008279 | arrest of spermiogenesis |
| MP:0008545 | absent sperm flagellum |
| MP:0008839 | absent acrosome |
| MP:0008892 | abnormal sperm flagellum morphology |
| MP:0008893 | detached sperm flagellum |
| MP:0008898 | abnormal acrosome morphology |
| MP:0009230 | abnormal sperm head morphology |
| MP:0009231 | detached acrosome |
| MP:0009232 | abnormal sperm nucleus morphology |
| MP:0009233 | enlarged sperm head |
| MP:0009234 | absent sperm head |
| MP:0009235 | small sperm head |
| MP:0009236 | pinhead sperm |
| MP:0009237 | kinked sperm flagellum |
| MP:0009238 | coiled sperm flagellum |
| MP:0009239 | short sperm flagellum |
| MP:0009240 | elongated sperm flagellum |
| MP:0009241 | thick sperm flagellum |
| MP:0009242 | thin sperm flagellum |
| MP:0009243 | hairpin sperm flagellum |
| MP:0009325 | necrospermia |
| MP:0009376 | abnormal manchette morphology |
| MP:0009377 | ectopic manchette |
| MP:0009830 | abnormal sperm connecting piece morphology |
| MP:0009831 | abnormal sperm midpiece morphology |
| MP:0009832 | abnormal sperm mitochondrial sheath morphology |
| MP:0009833 | absent sperm mitochondrial sheath |
| MP:0009834 | abnormal sperm annulus morphology |
| MP:0009835 | absent sperm annulus |
| MP:0009836 | abnormal sperm principal piece morphology |
| MP:0009837 | abnormal sperm end piece morphology |
| MP:0009838 | abnormal sperm axoneme morphology |
| MP:0009839 | multiflagellated sperm |
| MP:0009851 | abnormal Sertoli cell phagocytosis |
| MP:0009852 | increased Sertoli cell phagocytosis |
| MP:0009853 | decreased Sertoli cell phagocytosis |
| MP:0011751 | abnormal X-Y chromosome synapsis during male meiosis |
| MP:0011752 | abnormal X-Y chromosome synaptonemal complex assembly during male meiosis |
| MP:0002673 | abnormal sperm number |
| MP:0001378 | abnormal ejaculation |
| MP:0001379 | abnormal penile erection |
| MP:0001875 | testis inflammation |
| MP:0002674 | abnormal sperm motility |
| MP:0002675 | asthenozoospermia |
| MP:0002782 | abnormal testes secretion |
| MP:0003415 | priapism |
| MP:0003555 | chordee |
| MP:0003607 | abnormal prostate gland physiology |
| MP:0003608 | prostate gland inflammation |
| MP:0003666 | impaired sperm capacitation |
| MP:0003698 | abnormal male reproductive system physiology |
| MP:0004542 | impaired acrosome reaction |
| MP:0004543 | abnormal sperm physiology |
| MP:0004884 | abnormal testis physiology |
| MP:0008280 | abnormal male germ cell apoptosis |
| MP:0009279 | abnormal activated sperm motility |
| MP:0009280 | reduced activated sperm motility |
| MP:0009281 | abnormal hyperactivated sperm motility |
| MP:0009282 | reduced hyperactivated sperm motility |
| MP:0009856 | failure of ejaculation |
| MP:0011781 | abnormal bulbourethral gland physiology |
| MP:0013143 | penis inflammation |
| MP:0013286 | abnormal sperm capacitation |
| MP:0013287 | abnormal acrosome reaction |
| MP:0013288 | premature acrosome reaction |
| MP:0000659 | prostate gland hyperplasia |
| MP:0000660 | lateral prostate gland hypoplasia |
| MP:0000661 | small prostate gland ventral lobe |
| MP:0000664 | small prostate gland anterior lobe |
| MP:0000665 | decreased ductal branching in the coagulating gland |
| MP:0000666 | decreased prostate gland duct number |
| MP:0000671 | bulbourethral gland hypoplasia |
| MP:0001145 | abnormal male reproductive system morphology |
| MP:0001146 | abnormal testis morphology |
| MP:0001147 | small testis |
| MP:0001148 | enlarged testis |
| MP:0001149 | testicular hyperplasia |
| MP:0001150 | enlarged scrotum |
| MP:0001151 | enlarged foreskin |
| MP:0001152 | Leydig cell hyperplasia |
| MP:0001153 | small seminiferous tubules |
| MP:0001154 | seminiferous tubule degeneration |
| MP:0001157 | small seminal vesicle |
| MP:0001158 | abnormal prostate gland morphology |
| MP:0001159 | absent prostate gland |
| MP:0001163 | abnormal prostate gland anterior lobe morphology |
| MP:0001167 | prostate gland epithelial hyperplasia |
| MP:0001168 | abnormal prostate gland epithelium morphology |
| MP:0001169 | abnormal bulbourethral gland morphology |
| MP:0001170 | bulbourethral gland hyperplasia |
| MP:0001940 | testis hypoplasia |
| MP:0002059 | abnormal seminal vesicle morphology |
| MP:0002216 | abnormal seminiferous tubule morphology |
| MP:0002286 | cryptorchism |
| MP:0002631 | abnormal epididymis morphology |
| MP:0002660 | abnormal caput epididymis morphology |
| MP:0002661 | abnormal corpus epididymis morphology |
| MP:0002662 | abnormal cauda epididymis morphology |
| MP:0002669 | abnormal scrotum morphology |
| MP:0002670 | absent scrotum |
| MP:0002716 | small male preputial glands |
| MP:0002717 | abnormal male preputial gland morphology |
| MP:0002769 | abnormal vas deferens morphology |
| MP:0002770 | absent bulbourethral gland |
| MP:0002771 | absent prostate gland anterior lobe |
| MP:0002774 | small prostate gland |
| MP:0002776 | Sertoli cell hyperplasia |
| MP:0002784 | abnormal Sertoli cell morphology |
| MP:0002785 | absent Leydig cells |
| MP:0002786 | abnormal Leydig cell morphology |
| MP:0002997 | enlarged seminal vesicle |
| MP:0003205 | testicular atrophy |
| MP:0003435 | herniated seminal vesicle |
| MP:0003553 | abnormal foreskin morphology |
| MP:0003554 | phimosis |
| MP:0003557 | absent vas deferens |
| MP:0003595 | epididymal cyst |
| MP:0003598 | epispadia |
| MP:0003599 | large penis |
| MP:0003609 | small scrotum |
| MP:0003610 | scrotum hyperplasia |
| MP:0003611 | scrotum hypoplasia |
| MP:0003612 | bifid scrotum |
| MP:0003623 | hydrocele |
| MP:0003642 | absent seminal vesicle |
| MP:0003830 | abnormal testis development |
| MP:0004109 | abnormal Sertoli cell development |
| MP:0004727 | absent epididymis |
| MP:0004728 | abnormal efferent ductules of testis morphology |
| MP:0004729 | absent efferent ductules of testis |
| MP:0004849 | abnormal testis size |
| MP:0004850 | abnormal testis weight |
| MP:0004851 | increased testis weight |
| MP:0004852 | decreased testis weight |
| MP:0004907 | abnormal seminal vesicle size |
| MP:0004908 | abnormal seminal vesicle weight |
| MP:0004909 | increased seminal vesicle weight |
| MP:0004910 | decreased seminal vesicle weight |
| MP:0004926 | abnormal epididymis size |
| MP:0004927 | abnormal epididymis weight |
| MP:0004928 | increased epididymis weight |
| MP:0004929 | decreased epididymis weight |
| MP:0004930 | small epididymis |
| MP:0004931 | enlarged epididymis |
| MP:0004932 | epididymis hypoplasia |
| MP:0004933 | abnormal epididymis epithelium morphology |
| MP:0004934 | epididymis epithelium degeneration |
| MP:0004935 | epididymis degeneration |
| MP:0004958 | enlarged prostate gland |
| MP:0004959 | abnormal prostate gland size |
| MP:0004960 | abnormal prostate gland weight |
| MP:0004961 | increased prostate gland weight |
| MP:0004962 | decreased prostate gland weight |
| MP:0005147 | prostate gland hypoplasia |
| MP:0005148 | seminal vesicle hypoplasia |
| MP:0005187 | abnormal penis morphology |
| MP:0005188 | small penis |
| MP:0005250 | Sertoli cell hypoplasia |
| MP:0005304 | cystic bulbourethral gland |
| MP:0005305 | prostate gland anterior lobe hyperplasia |
| MP:0005536 | Leydig cell hypoplasia |
| MP:0006307 | abnormal seminiferous tubule size |
| MP:0006308 | enlarged seminiferous tubules |
| MP:0006401 | absent male preputial gland |
| MP:0006415 | absent testes |
| MP:0006416 | abnormal rete testis morphology |
| MP:0006417 | rete testis obstruction |
| MP:0006418 | abnormal testis cord formation |
| MP:0006419 | disorganized testis cords |
| MP:0006420 | abnormal peritubular myoid cell morphology |
| MP:0006421 | decreased number of peritubular myoid cells |
| MP:0006423 | dilated rete testis |
| MP:0006424 | absent testis cords |
| MP:0006427 | ectopic Leydig cells |
| MP:0006428 | ectopic Sertoli cells |
| MP:0008016 | abnormal male inguinal canal morphology |
| MP:0009102 | abnormal glans penis morphology |
| MP:0009103 | abnormal penile bone morphology |
| MP:0009104 | small penile bone |
| MP:0009105 | penis prolapse |
| MP:0009140 | dilated efferent ductules of testis |
| MP:0009198 | abnormal male genitalia morphology |
| MP:0009199 | abnormal external male genitalia morphology |
| MP:0009200 | enlarged external male genitalia |
| MP:0009201 | external male genitalia atrophy |
| MP:0009202 | small external male genitalia |
| MP:0009203 | external male genitalia hypoplasia |
| MP:0009204 | absent external male genitalia |
| MP:0009205 | abnormal internal male genitalia morphology |
| MP:0009206 | absent internal male genitalia |
| MP:0009207 | internal male genitalia hypoplasia |
| MP:0009213 | absent male inguinal canal |
| MP:0009214 | vas deferens hypoplasia |
| MP:0009248 | small caput epididymis |
| MP:0009249 | enlarged caput epididymis |
| MP:0009256 | enlarged corpus epididymis |
| MP:0009257 | dilated seminiferous tubules |
| MP:0009380 | abnormal prostate gland ventral lobe morphology |
| MP:0009381 | abnormal prostate gland dorsolateral lobe morphology |
| MP:0009734 | abnormal prostate gland duct morphology |
| MP:0009735 | abnormal prostate gland development |
| MP:0009736 | abnormal prostate gland branching morphogenesis |
| MP:0009737 | prostate gland cysts |
| MP:0009738 | enlarged prostate gland anterior lobe |
| MP:0009739 | enlarged prostate gland dorsolateral lobe |
| MP:0009740 | small prostate gland dorsolateral lobe |
| MP:0009824 | spermatic granuloma |
| MP:0009847 | abnormal scrotum pigmentation |
| MP:0010033 | paraphimosis |
| MP:0010145 | abnormal spermatic cord morphology |
| MP:0011410 | ectopic testis |
| MP:0011416 | abnormal testis interstitial tissue morphology |
| MP:0011750 | abnormal seminiferous tubule epithelium morphology |
| MP:0011876 | absent penis |
| MP:0013317 | abnormal seminal vesicle development |
| MP:0013318 | abnormal branching involved in seminal vesicle morphogenesis |
| MP:0013319 | seminal vesicle atrophy |
| MP:0013320 | dilated seminal vesicles |
| MP:0013321 | squamous metaplasia of seminal vesicles |
| MP:0013322 | squamous metaplasia of prostate gland |
| MP:0013323 | abnormal ampullary gland morphology |
| MP:0013598 | Leydig cell hypertrophy |
| MP:0013599 | Leydig cell atrophy |
| MP:0013600 | testis degeneration |
| MP:0013601 | increased testis apoptosis |
| MP:0013602 | abnormal Leydig cell differentiation |
| MP:0013603 | abnormal fetal Leydig cell differentiation |
| MP:0013604 | abnormal adult Leydig cell differentiation |
| MP:0013733 | squamous metaplasia of bulbourethral gland |
| MP:0013736 | abnormal bulbourethral gland development |
| MP:0013737 | small bulbourethral gland |
| MP:0013738 | abnormal testis tunica albuginea morphology |
| MP:0013739 | abnormal testis tunica vaginalis morphology |
| MP:0013327 | abnormal male reproductive gland physiology |
| MP:0011794 | abnormal male urethral gland morphology |
| MP:0013325 | abnormal male reproductive gland morphology |
| MP:0013330 | abnormal male accessory sex gland morphology |
| MP:0000242 | impaired fertilization |
| MP:0005410 | abnormal fertilization |
| MP:0005411 | delayed fertilization |
| MP:0009647 | decreased fertilization frequency |
| MP:0001116 | small gonad |
| MP:0001127 | small ovary |
| MP:0001937 | abnormal sexual maturation |
| MP:0001938 | delayed sexual maturation |
| MP:0001939 | secondary sex reversal |
| MP:0002210 | abnormal sex determination |
| MP:0002211 | abnormal primary sex determination |
| MP:0002212 | abnormal secondary sex determination |
| MP:0002213 | true hermaphroditism |
| MP:0002214 | streak gonad |
| MP:0002636 | delayed vaginal opening |
| MP:0002683 | delayed fertility |
| MP:0002787 | pseudohermaphroditism |
| MP:0002788 | female pseudohermaphroditism |
| MP:0002789 | male pseudohermaphroditism |
| MP:0002995 | primary sex reversal |
| MP:0002996 | ovotestis |
| MP:0003377 | late onset of menarche |
| MP:0003378 | early sexual maturation |
| MP:0003379 | absent sexual maturation |
| MP:0003578 | absent ovary |
| MP:0004833 | ovary atrophy |
| MP:0004856 | decreased ovary weight |
| MP:0005158 | ovary hypoplasia |
| MP:0005644 | agonadal |
| MP:0005652 | sex reversal |
| MP:0008940 | delayed balanopreputial separation |
| MP:0008975 | delayed male fertility |
| MP:0008976 | delayed female fertility |
| MP:0008994 | early vaginal opening |
| MP:0020150 | abnormal timing of vaginal opening |
| MP:0004901 | decreased male germ cell number |
| MP:0006362 | abnormal male germ cell morphology |
| MP:0006378 | abnormal spermatogonia morphology |
| MP:0001924 | infertility |
| MP:0001921 | reduced fertility |
| MP:0002161 | abnormal fertility/fecundity |
| MP:0001935 | decreased litter size^c^ |

^a^MP, mammalian phenotype; ^b^If literature was given in MGI files, phenotypes were checked in the quoted articles. Only genes linked to phenotypes which result in male sub- or infertility were included in the infertility category. If a phenotype potentially applying to both sexes (e.g. “infertility”) was found to be associated with female reproductive abnormalities upon knockout, the corresponding gene was categorized as nonessential / lethal (see Materials and Methods of the main text). The same applies to all female phenotypes included as parts of higher order MP IDs such as “abnormal sex determination” (MP:0002210). ^c^For “decreased litter size” (MP:0001935), see Materials and Methods section of the main text.

**Supplementary References**

1. Chapple, C. E. *et al.* Extreme multifunctional proteins identified from a human protein interaction network. *Nat Commun* **6,** 7412 (2015).

2. Kryuchkova-Mostacci, N. & Robinson-Rechavi, M. Tissue-specific evolution of protein coding genes in human and mouse. *PLoS One* **10,** e0131673 (2015).

3. Fagerberg, L. *et al.* Analysis of the human tissue-specific expression by genome-wide integration of transcriptomics and antibody-based proteomics. *Mol Cell Proteomics* **13**, 397–406 (2014).
